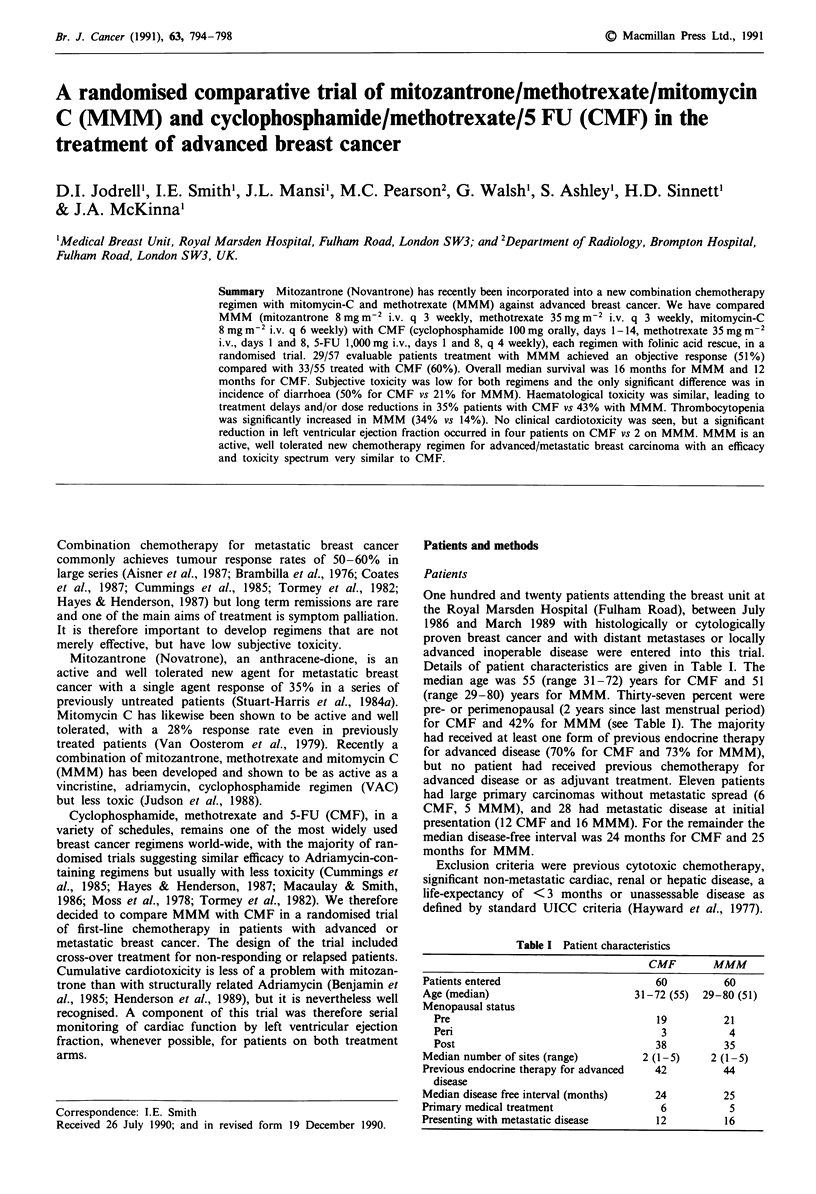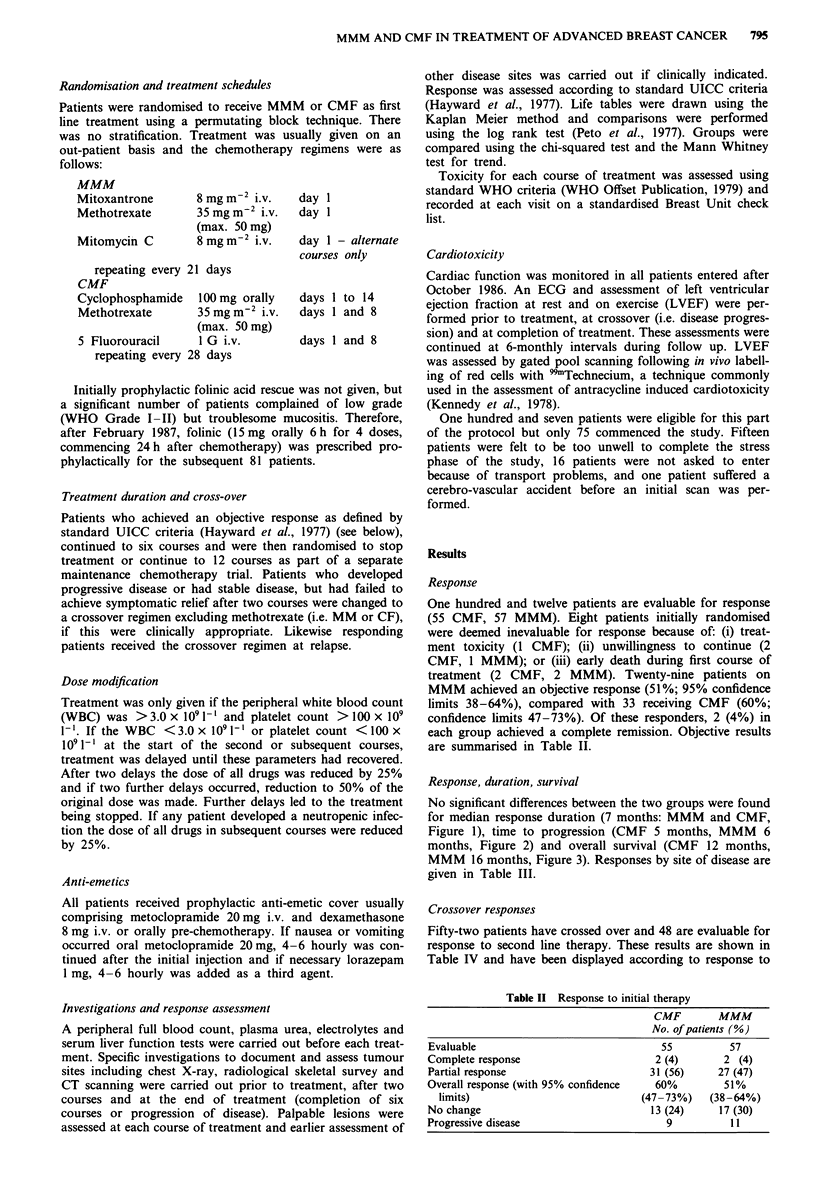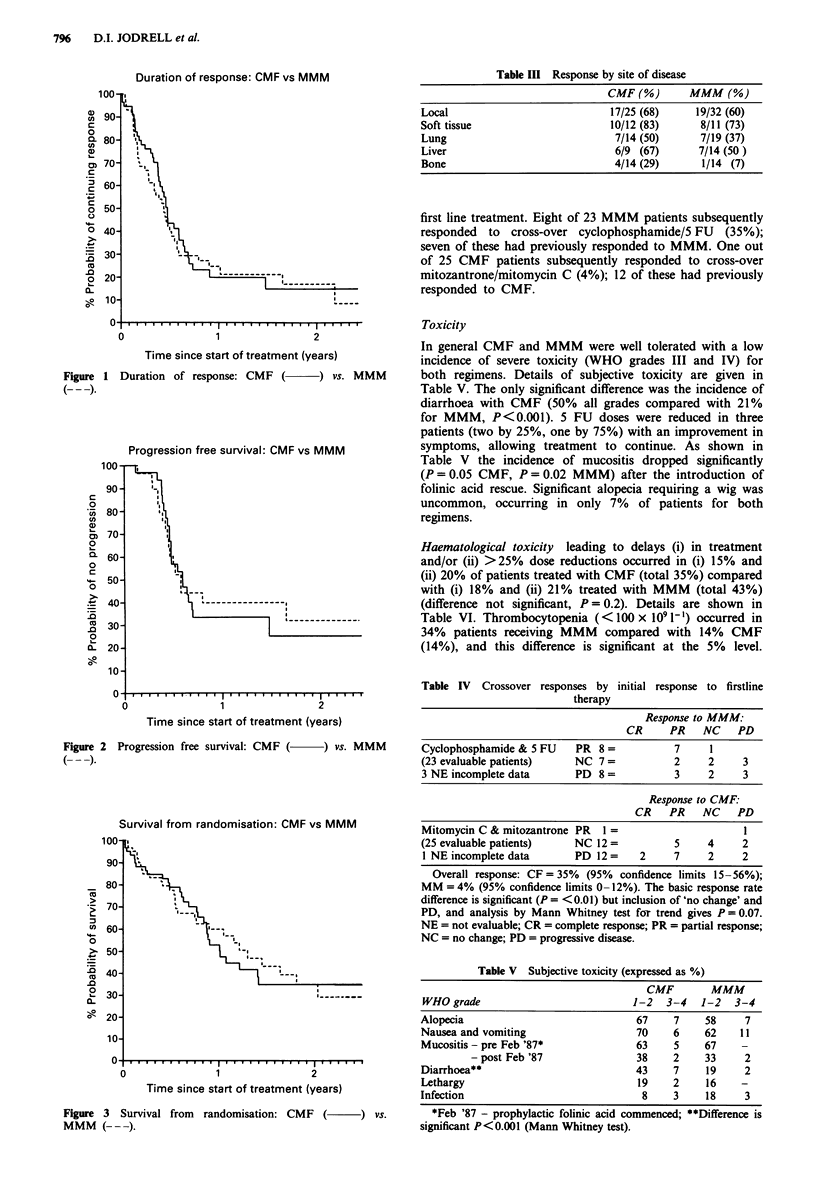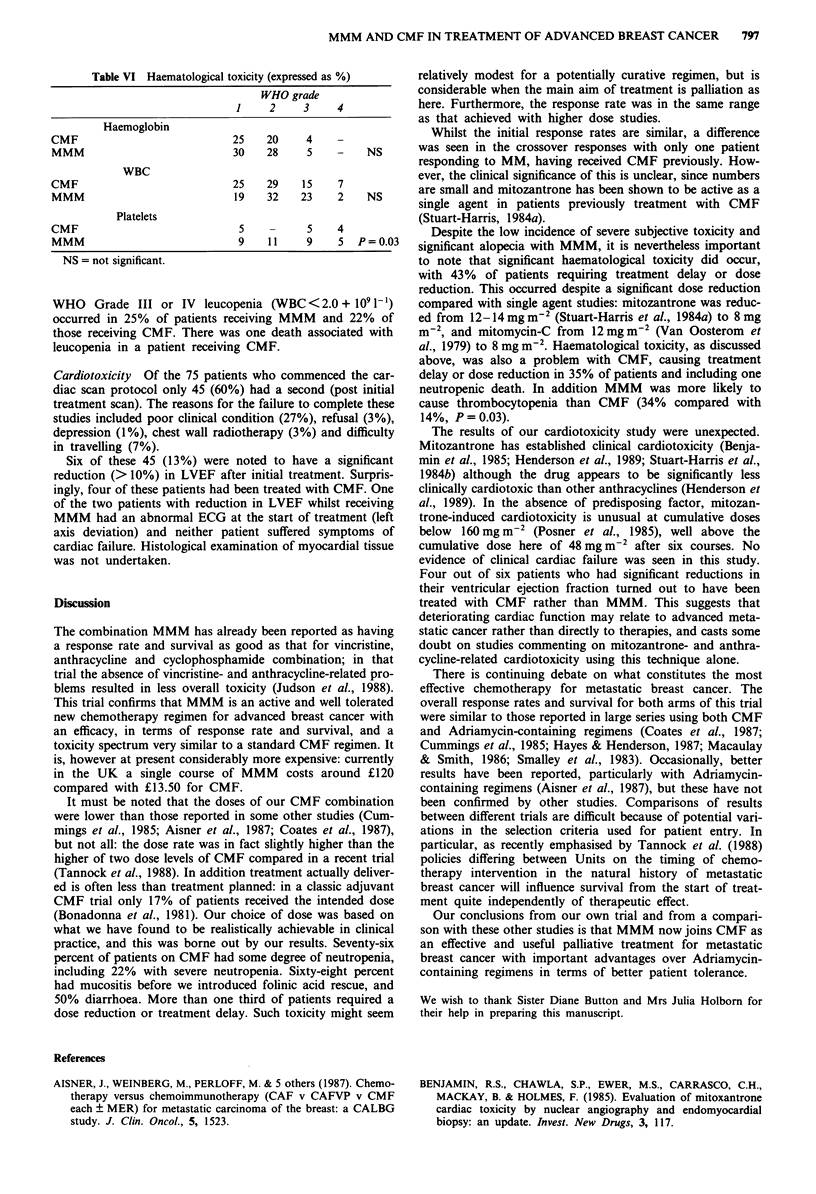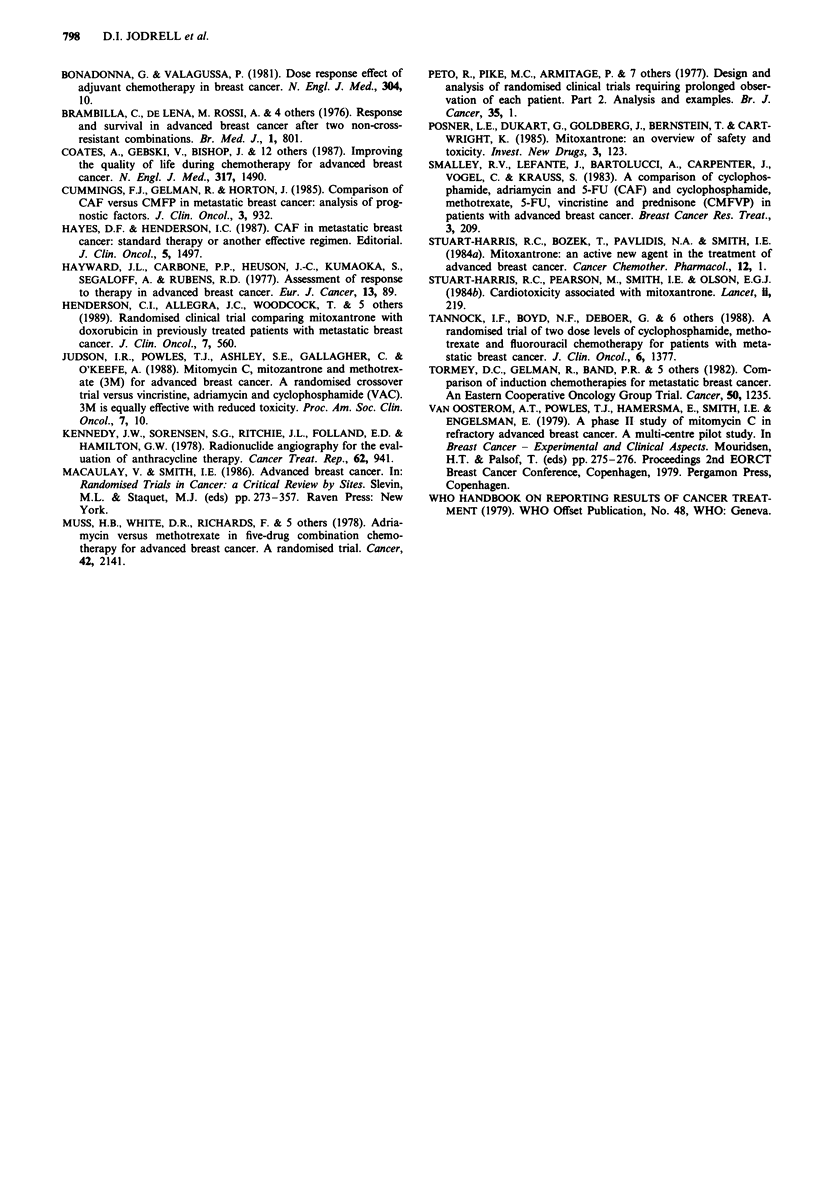# A randomised comparative trial of mitozantrone/methotrexate/mitomycin C (MMM) and cyclophosphamide/methotrexate/5 FU (CMF) in the treatment of advanced breast cancer.

**DOI:** 10.1038/bjc.1991.176

**Published:** 1991-05

**Authors:** D. I. Jodrell, I. E. Smith, J. L. Mansi, M. C. Pearson, G. Walsh, S. Ashley, H. D. Sinnett, J. A. McKinna

**Affiliations:** Medical Breast Unit, Royal Marsden Hospital, London, UK.

## Abstract

Mitozantrone (Novantrone) has recently been incorporated into a new combination chemotherapy regimen with mitomycin-C and methotrexate (MMM) against advanced breast cancer. We have compared MMM (mitozantrone 8 mg m-2 i.v. q 3 weekly, methotrexate 35 mg m-2 i.v. q 3 weekly, mitomycin-C 8 mg m-2 i.v. q 6 weekly) with CMF (cyclophosphamide 100 mg orally, days 1-14, methotrexate 35 mg m-2 i.v., days 1 and 8, 5-FU 1,000 mg i.v., days 1 and 8, q 4 weekly), each regimen with folinic acid rescue, in a randomised trial, 29/57 evaluable patients treatment with MMM achieved an objective response (51%) compared with 33/55 treated with CMF (60%). Overall median survival was 16 months for MMM and 12 months for CMF. Subjective toxicity was low for both regimens and the only significant difference was in incidence of diarrhoea (50% for CMF vs 21% for MMM). Haematological toxicity was similar, leading to treatment delays and/or dose reductions in 35% patients with CMF vs 43% with MMM. Thrombocytopenia was significantly increased in MMM (34% vs 14%). No clinical cardiotoxicity was seen, but a significant reduction in left ventricular ejection fraction occurred in four patients on CMF vs 2 on MMM. MMM is an active, well tolerated new chemotherapy regimen for advanced/metastatic breast carcinoma with an efficacy and toxicity spectrum very similar to CMF.


Br. J. Cancer (1991), 63, 794–798 © Macmillan Press Ltd., 1991

### A randomised comparative trial of mitozantrone/methotrexate/mitomycin C (MMM) and cyclophosphamide/methotrexate/5 FU (CMF) in the treatment of advanced breast cancer

D.I. Jodrell[1], I.E. Smith[1], J.L. Mansi[1], M.C. Pearson[2], G. Walsh[1], S. Ashley[1], H.D. Sinnett[1] & J.A. McKinna[1]

[1]*Medical Breast Unit, Royal Marsden Hospital, Fulham Road, London SW3; and* [2]*Department of Radiology, Brompton Hospital, Fulham Road, London SW3, UK.*

**Summary** Mitozantrone (Novantrone) has recently been incorporated into a new combination chemotherapy regimen with mitomycin-C and methotrexate (MMM) against advanced breast cancer. We have compared MMM (mitozantrone 8 mg $m^{-2}$ i.v. q 3 weekly, methotrexate 35 mg $m^{-2}$ i.v. q 3 weekly, mitomycin-C 8 mg $m^{-2}$ i.v. q 6 weekly) with CMF (cyclophosphamide 100 mg orally, days 1–14, methotrexate 35 mg $m^{-2}$ i.v., days 1 and 8, 5-FU 1,000 mg i.v., days 1 and 8, q 4 weekly), each regimen with folinic acid rescue, in a randomised trial. 29/57 evaluable patients treatment with MMM achieved an objective response (51%) compared with 33/55 treated with CMF (60%). Overall median survival was 16 months for MMM and 12 months for CMF. Subjective toxicity was low for both regimens and the only significant difference was in incidence of diarrhoea (50% for CMF *vs* 21% for MMM). Haematological toxicity was similar, leading to treatment delays and/or dose reductions in 35% patients with CMF *vs* 43% with MMM. Thrombocytopenia was significantly increased in MMM (34% *vs* 14%). No clinical cardiotoxicity was seen, but a significant reduction in left ventricular ejection fraction occurred in four patients on CMF *vs* 2 on MMM. MMM is an active, well tolerated new chemotherapy regimen for advanced/metastatic breast carcinoma with an efficacy and toxicity spectrum very similar to CMF.

Combination chemotherapy for metastatic breast cancer commonly achieves tumour response rates of 50–60% in large series (Aisner *et al.*, 1987; Brambilla *et al.*, 1976; Coates *et al.*, 1987; Cummings *et al.*, 1985; Tormey *et al.*, 1982; Hayes & Henderson, 1987) but long term remissions are rare and one of the main aims of treatment is symptom palliation. It is therefore important to develop regimens that are not merely effective, but have low subjective toxicity.

Mitozantrone (Novatrone), an anthracene-dione, is an active and well tolerated new agent for metastatic breast cancer with a single agent response of 35% in a series of previously untreated patients (Stuart-Harris *et al.*, 1984*a*). Mitomycin C has likewise been shown to be active and well tolerated, with a 28% response rate even in previously treated patients (Van Oosterom *et al.*, 1979). Recently a combination of mitozantrone, methotrexate and mitomycin C (MMM) has been developed and shown to be as active as a vincristine, adriamycin, cyclophosphamide regimen (VAC) but less toxic (Judson *et al.*, 1988).

Cyclophosphamide, methotrexate and 5-FU (CMF), in a variety of schedules, remains one of the most widely used breast cancer regimens world-wide, with the majority of randomised trials suggesting similar efficacy to Adriamycin-containing regimens but usually with less toxicity (Cummings *et al.*, 1985; Hayes & Henderson, 1987; Macaulay & Smith, 1986; Moss *et al.*, 1978; Tormey *et al.*, 1982). We therefore decided to compare MMM with CMF in a randomised trial of first-line chemotherapy in patients with advanced or metastatic breast cancer. The design of the trial included cross-over treatment for non-responding or relapsed patients. Cumulative cardiotoxicity is less of a problem with mitozantrone than with structurally related Adriamycin (Benjamin *et al.*, 1985; Henderson *et al.*, 1989), but it is nevertheless well recognised. A component of this trial was therefore serial monitoring of cardiac function by left ventricular ejection fraction, whenever possible, for patients on both treatment arms.

Correspondence: I.E. Smith
Received 26 July 1990; and in revised form 19 December 1990.

### Patients and methods

### *Patients*

One hundred and twenty patients attending the breast unit at the Royal Marsden Hospital (Fulham Road), between July 1986 and March 1989 with histologically or cytologically proven breast cancer and with distant metastases or locally advanced inoperable disease were entered into this trial. Details of patient characteristics are given in Table I. The median age was 55 (range 31–72) years for CMF and 51 (range 29–80) years for MMM. Thirty-seven percent were pre- or perimenopausal (2 years since last menstrual period) for CMF and 42% for MMM (see Table I). The majority had received at least one form of previous endocrine therapy for advanced disease (70% for CMF and 73% for MMM), but no patient had received previous chemotherapy for advanced disease or as adjuvant treatment. Eleven patients had large primary carcinomas without metastatic spread (6 CMF, 5 MMM), and 28 had metastatic disease at initial presentation (12 CMF and 16 MMM). For the remainder the median disease-free interval was 24 months for CMF and 25 months for MMM.

Exclusion criteria were previous cytotoxic chemotherapy, significant non-metastatic cardiac, renal or hepatic disease, a life-expectancy of <3 months or unassessable disease as defined by standard UICC criteria (Hayward *et al.*, 1977).

**Table I** Patient characteristics

| | *CMF* | *MMM* |
|---|---|---|
| Patients entered | 60 | 60 |
| Age (median) | 31–72 (55) | 29–80 (51) |
| Menopausal status | | |
| Pre | 19 | 21 |
| Peri | 3 | 4 |
| Post | 38 | 35 |
| Median number of sites (range) | 2 (1–5) | 2 (1–5) |
| Previous endocrine therapy for advanced disease | 42 | 44 |
| Median disease free interval (months) | 24 | 25 |
| Primary medical treatment | 6 | 5 |
| Presenting with metastatic disease | 12 | 16 |

*Randomisation and treatment schedules*

Patients were randomised to receive MMM or CMF as first line treatment using a permutating block technique. There was no stratification. Treatment was usually given on an out-patient basis and the chemotherapy regimens were as follows:

| | | |
|---|---|---|
| *MMM* | | |
| Mitoxantrone | 8 mg $m^{-2}$ i.v. | day 1 |
| Methotrexate | 35 mg $m^{-2}$ i.v. (max. 50 mg) | day 1 |
| Mitomycin C | 8 mg $m^{-2}$ i.v. | day 1 – *alternate courses only* |
| repeating every 21 days | | |
| *CMF* | | |
| Cyclophosphamide | 100 mg orally | days 1 to 14 |
| Methotrexate | 35 mg $m^{-2}$ i.v. (max. 50 mg) | days 1 and 8 |
| 5 Fluorouracil | 1 G i.v. | days 1 and 8 |
| repeating every 28 days | | |

Initially prophylactic folinic acid rescue was not given, but a significant number of patients complained of low grade (WHO Grade I–II) but troublesome mucositis. Therefore, after February 1987, folinic (15 mg orally 6 h for 4 doses, commencing 24 h after chemotherapy) was prescribed prophylactically for the subsequent 81 patients.

*Treatment duration and cross-over*

Patients who achieved an objective response as defined by standard UICC criteria (Hayward *et al.*, 1977) (see below), continued to six courses and were then randomised to stop treatment or continue to 12 courses as part of a separate maintenance chemotherapy trial. Patients who developed progressive disease or had stable disease, but had failed to achieve symptomatic relief after two courses were changed to a crossover regimen excluding methotrexate (i.e. MM or CF), if this were clinically appropriate. Likewise responding patients received the crossover regimen at relapse.

*Dose modification*

Treatment was only given if the peripheral white blood count (WBC) was $>3.0 \times 10^9 l^{-1}$ and platelet count $>100 \times 10^9 l^{-1}$. If the WBC $<3.0 \times 10^9 l^{-1}$ or platelet count $<100 \times 10^9 l^{-1}$ at the start of the second or subsequent courses, treatment was delayed until these parameters had recovered. After two delays the dose of all drugs was reduced by 25% and if two further delays occurred, reduction to 50% of the original dose was made. Further delays led to the treatment being stopped. If any patient developed a neutropenic infection the dose of all drugs in subsequent courses were reduced by 25%.

*Anti-emetics*

All patients received prophylactic anti-emetic cover usually comprising metoclopramide 20 mg i.v. and dexamethasone 8 mg i.v. or orally pre-chemotherapy. If nausea or vomiting occurred oral metoclopramide 20 mg, 4–6 hourly was continued after the initial injection and if necessary lorazepam 1 mg, 4–6 hourly was added as a third agent.

*Investigations and response assessment*

A peripheral full blood count, plasma urea, electrolytes and serum liver function tests were carried out before each treatment. Specific investigations to document and assess tumour sites including chest X-ray, radiological skeletal survey and CT scanning were carried out prior to treatment, after two courses and at the end of treatment (completion of six courses or progression of disease). Palpable lesions were assessed at each course of treatment and earlier assessment of other disease sites was carried out if clinically indicated. Response was assessed according to standard UICC criteria (Hayward *et al.*, 1977). Life tables were drawn using the Kaplan Meier method and comparisons were performed using the log rank test (Peto *et al.*, 1977). Groups were compared using the chi-squared test and the Mann Whitney test for trend.

Toxicity for each course of treatment was assessed using standard WHO criteria (WHO Offset Publication, 1979) and recorded at each visit on a standardised Breast Unit check list.

*Cardiotoxicity*

Cardiac function was monitored in all patients entered after October 1986. An ECG and assessment of left ventricular ejection fraction at rest and on exercise (LVEF) were performed prior to treatment, at crossover (i.e. disease progression) and at completion of treatment. These assessments were continued at 6-monthly intervals during follow up. LVEF was assessed by gated pool scanning following *in vivo* labelling of red cells with $^{99m}$Technecium, a technique commonly used in the assessment of antracycline induced cardiotoxicity (Kennedy *et al.*, 1978).

One hundred and seven patients were eligible for this part of the protocol but only 75 commenced the study. Fifteen patients were felt to be too unwell to complete the stress phase of the study, 16 patients were not asked to enter because of transport problems, and one patient suffered a cerebro-vascular accident before an initial scan was performed.

### Results

*Response*

One hundred and twelve patients are evaluable for response (55 CMF, 57 MMM). Eight patients initially randomised were deemed inevaluable for response because of: (i) treatment toxicity (1 CMF); (ii) unwillingness to continue (2 CMF, 1 MMM); or (iii) early death during first course of treatment (2 CMF, 2 MMM). Twenty-nine patients on MMM achieved an objective response (51%; 95% confidence limits 38–64%), compared with 33 receiving CMF (60%; confidence limits 47–73%). Of these responders, 2 (4%) in each group achieved a complete remission. Objective results are summarised in Table II.

*Response, duration, survival*

No significant differences between the two groups were found for median response duration (7 months: MMM and CMF, Figure 1), time to progression (CMF 5 months, MMM 6 months, Figure 2) and overall survival (CMF 12 months, MMM 16 months, Figure 3). Responses by site of disease are given in Table III.

*Crossover responses*

Fifty-two patients have crossed over and 48 are evaluable for response to second line therapy. These results are shown in Table IV and have been displayed according to response to

**Table II** Response to initial therapy

| | *CMF* | *MMM* |
|---|---|---|
| | *No. of patients (%)* | |
| Evaluable | 55 | 57 |
| Complete response | 2 (4) | 2 (4) |
| Partial response | 31 (56) | 27 (47) |
| Overall response (with 95% confidence limits) | 60% (47–73%) | 51% (38–64%) |
| No change | 13 (24) | 17 (30) |
| Progressive disease | 9 | 11 |

**Figure 1** Duration of response: CMF (———) vs. MMM (– – –).

**Figure 2** Progression free survival: CMF (———) vs. MMM (– – –).

**Figure 3** Survival from randomisation: CMF (———) vs. MMM (– – –).

**Table III** Response by site of disease

| | *CMF (%)* | *MMM (%)* |
|---|---|---|
| Local | 17/25 (68) | 19/32 (60) |
| Soft tissue | 10/12 (83) | 8/11 (73) |
| Lung | 7/14 (50) | 7/19 (37) |
| Liver | 6/9 (67) | 7/14 (50 ) |
| Bone | 4/14 (29) | 1/14 (7) |

first line treatment. Eight of 23 MMM patients subsequently responded to cross-over cyclophosphamide/5 FU (35%); seven of these had previously responded to MMM. One out of 25 CMF patients subsequently responded to cross-over mitozantrone/mitomycin C (4%); 12 of these had previously responded to CMF.

*Toxicity*

In general CMF and MMM were well tolerated with a low incidence of severe toxicity (WHO grades III and IV) for both regimens. Details of subjective toxicity are given in Table V. The only significant difference was the incidence of diarrhoea with CMF (50% all grades compared with 21% for MMM, $P<0.001$). 5 FU doses were reduced in three patients (two by 25%, one by 75%) with an improvement in symptoms, allowing treatment to continue. As shown in Table V the incidence of mucositis dropped significantly ($P=0.05$ CMF, $P=0.02$ MMM) after the introduction of folinic acid rescue. Significant alopecia requiring a wig was uncommon, occurring in only 7% of patients for both regimens.

*Haematological toxicity* leading to delays (i) in treatment and/or (ii) >25% dose reductions occurred in (i) 15% and (ii) 20% of patients treated with CMF (total 35%) compared with (i) 18% and (ii) 21% treated with MMM (total 43%) (difference not significant, $P=0.2$). Details are shown in Table VI. Thrombocytopenia ($<100\times10^9\,l^{-1}$) occurred in 34% patients receiving MMM compared with 14% CMF (14%), and this difference is significant at the 5% level.

**Table IV** Crossover responses by initial response to firstline therapy

| | | *Response to MMM:* | | | |
|---|---|---|---|---|---|
| | | *CR* | *PR* | *NC* | *PD* |
| Cyclophosphamide & 5 FU | PR 8 = | | 7 | 1 | |
| (23 evaluable patients) | NC 7 = | | 2 | 2 | 3 |
| 3 NE incomplete data | PD 8 = | | 3 | 2 | 3 |
| | | *Response to CMF:* | | | |
| | | *CR* | *PR* | *NC* | *PD* |
| Mitomycin C & mitozantrone | PR 1 = | | | | 1 |
| (25 evaluable patients) | NC 12 = | | 5 | 4 | 2 |
| 1 NE incomplete data | PD 12 = | 2 | 7 | 2 | 2 |

Overall response: CF = 35% (95% confidence limits 15–56%); MM = 4% (95% confidence limits 0–12%). The basic response rate difference is significant ($P=<0.01$) but inclusion of 'no change' and PD, and analysis by Mann Whitney test for trend gives $P=0.07$. NE = not evaluable; CR = complete response; PR = partial response; NC = no change; PD = progressive disease.

**Table V** Subjective toxicity (expressed as %)

| | *CMF* | | *MMM* | |
|---|---|---|---|---|
| *WHO grade* | *1–2* | *3–4* | *1–2* | *3–4* |
| Alopecia | 67 | 7 | 58 | 7 |
| Nausea and vomiting | 70 | 6 | 62 | 11 |
| Mucositis – pre Feb '87* | 63 | 5 | 67 | – |
| – post Feb '87 | 38 | 2 | 33 | 2 |
| Diarrhoea** | 43 | 7 | 19 | 2 |
| Lethargy | 19 | 2 | 16 | – |
| Infection | 8 | 3 | 18 | 3 |

*Feb '87 – prophylactic folinic acid commenced; **Difference is significant $P<0.001$ (Mann Whitney test).

**Table VI** Haematological toxicity (expressed as %)

| | WHO grade 1 | 2 | 3 | 4 | |
|---|---|---|---|---|---|
| Haemoglobin | | | | | |
| CMF | 25 | 20 | 4 | – | |
| MMM | 30 | 28 | 5 | – | NS |
| WBC | | | | | |
| CMF | 25 | 29 | 15 | 7 | |
| MMM | 19 | 32 | 23 | 2 | NS |
| Platelets | | | | | |
| CMF | 5 | – | 5 | 4 | |
| MMM | 9 | 11 | 9 | 5 | $P = 0.03$ |

NS = not significant.

WHO Grade III or IV leucopenia (WBC $< 2.0 + 10^9 l^{-1}$) occurred in 25% of patients receiving MMM and 22% of those receiving CMF. There was one death associated with leucopenia in a patient receiving CMF.

*Cardiotoxicity* Of the 75 patients who commenced the cardiac scan protocol only 45 (60%) had a second (post initial treatment scan). The reasons for the failure to complete these studies included poor clinical condition (27%), refusal (3%), depression (1%), chest wall radiotherapy (3%) and difficulty in travelling (7%).

Six of these 45 (13%) were noted to have a significant reduction (> 10%) in LVEF after initial treatment. Surprisingly, four of these patients had been treated with CMF. One of the two patients with reduction in LVEF whilst receiving MMM had an abnormal ECG at the start of treatment (left axis deviation) and neither patient suffered symptoms of cardiac failure. Histological examination of myocardial tissue was not undertaken.

### Discussion

The combination MMM has already been reported as having a response rate and survival as good as that for vincristine, anthracycline and cyclophosphamide combination; in that trial the absence of vincristine- and anthracycline-related problems resulted in less overall toxicity (Judson *et al.*, 1988). This trial confirms that MMM is an active and well tolerated new chemotherapy regimen for advanced breast cancer with an efficacy, in terms of response rate and survival, and a toxicity spectrum very similar to a standard CMF regimen. It is, however at present considerably more expensive: currently in the UK a single course of MMM costs around £120 compared with £13.50 for CMF.

It must be noted that the doses of our CMF combination were lower than those reported in some other studies (Cummings *et al.*, 1985; Aisner *et al.*, 1987; Coates *et al.*, 1987), but not all: the dose rate was in fact slightly higher than the higher of two dose levels of CMF compared in a recent trial (Tannock *et al.*, 1988). In addition treatment actually delivered is often less than treatment planned: in a classic adjuvant CMF trial only 17% of patients received the intended dose (Bonadonna *et al.*, 1981). Our choice of dose was based on what we have found to be realistically achievable in clinical practice, and this was borne out by our results. Seventy-six percent of patients on CMF had some degree of neutropenia, including 22% with severe neutropenia. Sixty-eight percent had mucositis before we introduced folinic acid rescue, and 50% diarrhoea. More than one third of patients required a dose reduction or treatment delay. Such toxicity might seem relatively modest for a potentially curative regimen, but is considerable when the main aim of treatment is palliation as here. Furthermore, the response rate was in the same range as that achieved with higher dose studies.

Whilst the initial response rates are similar, a difference was seen in the crossover responses with only one patient responding to MM, having received CMF previously. However, the clinical significance of this is unclear, since numbers are small and mitozantrone has been shown to be active as a single agent in patients previously treatment with CMF (Stuart-Harris, 1984*a*).

Despite the low incidence of severe subjective toxicity and significant alopecia with MMM, it is nevertheless important to note that significant haematological toxicity did occur, with 43% of patients requiring treatment delay or dose reduction. This occurred despite a significant dose reduction compared with single agent studies: mitozantrone was reduced from 12–14 mg m$^{-2}$ (Stuart-Harris *et al.*, 1984*a*) to 8 mg m$^{-2}$, and mitomycin-C from 12 mg m$^{-2}$ (Van Oosterom *et al.*, 1979) to 8 mg m$^{-2}$. Haematological toxicity, as discussed above, was also a problem with CMF, causing treatment delay or dose reduction in 35% of patients and including one neutropenic death. In addition MMM was more likely to cause thrombocytopenia than CMF (34% compared with 14%, $P = 0.03$).

The results of our cardiotoxicity study were unexpected. Mitozantrone has established clinical cardiotoxicity (Benjamin *et al.*, 1985; Henderson *et al.*, 1989; Stuart-Harris *et al.*, 1984*b*) although the drug appears to be significantly less clinically cardiotoxic than other anthracyclines (Henderson *et al.*, 1989). In the absence of predisposing factor, mitozantrone-induced cardiotoxicity is unusual at cumulative doses below 160 mg m$^{-2}$ (Posner *et al.*, 1985), well above the cumulative dose here of 48 mg m$^{-2}$ after six courses. No evidence of clinical cardiac failure was seen in this study. Four out of six patients who had significant reductions in their ventricular ejection fraction turned out to have been treated with CMF rather than MMM. This suggests that deteriorating cardiac function may relate to advanced metastatic cancer rather than directly to therapies, and casts some doubt on studies commenting on mitozantrone- and anthracycline-related cardiotoxicity using this technique alone.

There is continuing debate on what constitutes the most effective chemotherapy for metastatic breast cancer. The overall response rates and survival for both arms of this trial were similar to those reported in large series using both CMF and Adriamycin-containing regimens (Coates *et al.*, 1987; Cummings *et al.*, 1985; Hayes & Henderson, 1987; Macaulay & Smith, 1986; Smalley *et al.*, 1983). Occasionally, better results have been reported, particularly with Adriamycin-containing regimens (Aisner *et al.*, 1987), but these have not been confirmed by other studies. Comparisons of results between different trials are difficult because of potential variations in the selection criteria used for patient entry. In particular, as recently emphasised by Tannock *et al.* (1988) policies differing between Units on the timing of chemotherapy intervention in the natural history of metastatic breast cancer will influence survival from the start of treatment quite independently of therapeutic effect.

Our conclusions from our own trial and from a comparison with these other studies is that MMM now joins CMF as an effective and useful palliative treatment for metastatic breast cancer with important advantages over Adriamycin-containing regimens in terms of better patient tolerance.

We wish to thank Sister Diane Button and Mrs Julia Holborn for their help in preparing this manuscript.

### References

AISNER, J., WEINBERG, M., PERLOFF, M. & 5 others (1987). Chemotherapy versus chemoimmunotherapy (CAF v CAFVP v CMF each ± MER) for metastatic carcinoma of the breast: a CALBG study. *J. Clin. Oncol.*, **5**, 1523.

BENJAMIN, R.S., CHAWLA, S.P., EWER, M.S., CARRASCO, C.H., MACKAY, B. & HOLMES, F. (1985). Evaluation of mitoxantrone cardiac toxicity by nuclear angiography and endomyocardial biopsy: an update. *Invest. New Drugs*, **3**, 117.

BONADONNA, G. & VALAGUSSA, P. (1981). Dose response effect of adjuvant chemotherapy in breast cancer. *N. Engl. J. Med.*, **304**, 10.

BRAMBILLA, C., DE LENA, M. ROSSI, A. & 4 others (1976). Response and survival in advanced breast cancer after two non-cross-resistant combinations. *Br. Med. J.*, **1**, 801.

COATES, A., GEBSKI, V., BISHOP, J. & 12 others (1987). Improving the quality of life during chemotherapy for advanced breast cancer. *N. Engl. J. Med.*, **317**, 1490.

CUMMINGS, F.J., GELMAN, R. & HORTON, J. (1985). Comparison of CAF versus CMFP in metastatic breast cancer: analysis of prognostic factors. *J. Clin. Oncol.*, **3**, 932.

HAYES, D.F. & HENDERSON, I.C. (1987). CAF in metastatic breast cancer: standard therapy or another effective regimen. Editorial. *J. Clin. Oncol.*, **5**, 1497.

HAYWARD, J.L., CARBONE, P.P., HEUSON, J.-C., KUMAOKA, S., SEGALOFF, A. & RUBENS, R.D. (1977). Assessment of response to therapy in advanced breast cancer. *Eur. J. Cancer*, **13**, 89.

HENDERSON, C.I., ALLEGRA, J.C., WOODCOCK, T. & 5 others (1989). Randomised clinical trial comparing mitoxantrone with doxorubicin in previously treated patients with metastatic breast cancer. *J. Clin. Oncol.*, **7**, 560.

JUDSON, I.R., POWLES, T.J., ASHLEY, S.E., GALLAGHER, C. & O'KEEFE, A. (1988). Mitomycin C, mitozantrone and methotrexate (3M) for advanced breast cancer. A randomised crossover trial versus vincristine, adriamycin and cyclophosphamide (VAC). 3M is equally effective with reduced toxicity. *Proc. Am. Soc. Clin. Oncol.*, **7**, 10.

KENNEDY, J.W., SORENSEN, S.G., RITCHIE, J.L., FOLLAND, E.D. & HAMILTON, G.W. (1978). Radionuclide angiography for the evaluation of anthracycline therapy. *Cancer Treat. Rep.*, **62**, 941.

MACAULAY, V. & SMITH, I.E. (1986). Advanced breast cancer. In: *Randomised Trials in Cancer: a Critical Review by Sites*. Slevin, M.L. & Staquet, M.J. (eds) pp. 273–357. Raven Press: New York.

MUSS, H.B., WHITE, D.R., RICHARDS, F. & 5 others (1978). Adriamycin versus methotrexate in five-drug combination chemotherapy for advanced breast cancer. A randomised trial. *Cancer*, **42**, 2141.

PETO, R., PIKE, M.C., ARMITAGE, P. & 7 others (1977). Design and analysis of randomised clinical trials requiring prolonged observation of each patient. Part 2. Analysis and examples. *Br. J. Cancer*, **35**, 1.

POSNER, L.E., DUKART, G., GOLDBERG, J., BERNSTEIN, T. & CARTWRIGHT, K. (1985). Mitoxantrone: an overview of safety and toxicity. *Invest. New Drugs*, **3**, 123.

SMALLEY, R.V., LEFANTE, J., BARTOLUCCI, A., CARPENTER, J., VOGEL, C. & KRAUSS, S. (1983). A comparison of cyclophosphamide, adriamycin and 5-FU (CAF) and cyclophosphamide, methotrexate, 5-FU, vincristine and prednisone (CMFVP) in patients with advanced breast cancer. *Breast Cancer Res. Treat.*, **3**, 209.

STUART-HARRIS, R.C., BOZEK, T., PAVLIDIS, N.A. & SMITH, I.E. (1984*a*). Mitoxantrone: an active new agent in the treatment of advanced breast cancer. *Cancer Chemother. Pharmacol.*, **12**, 1.

STUART-HARRIS, R.C., PEARSON, M., SMITH, I.E. & OLSON, E.G.J. (1984*b*). Cardiotoxicity associated with mitoxantrone. *Lancet*, **ii**, 219.

TANNOCK, I.F., BOYD, N.F., DEBOER, G. & 6 others (1988). A randomised trial of two dose levels of cyclophosphamide, methotrexate and fluorouracil chemotherapy for patients with metastatic breast cancer. *J. Clin. Oncol.*, **6**, 1377.

TORMEY, D.C., GELMAN, R., BAND, P.R. & 5 others (1982). Comparison of induction chemotherapies for metastatic breast cancer. An Eastern Cooperative Oncology Group Trial. *Cancer*, **50**, 1235.

VAN OOSTEROM, A.T., POWLES, T.J., HAMERSMA, E., SMITH, I.E. & ENGELSMAN, E. (1979). A phase II study of mitomycin C in refractory advanced breast cancer. A multi-centre pilot study. In *Breast Cancer – Experimental and Clinical Aspects*. Mouridsen, H.T. & Palsof, T. (eds) pp. 275–276. Proceedings 2nd EORCT Breast Cancer Conference, Copenhagen, 1979. Pergamon Press, Copenhagen.

WHO HANDBOOK ON REPORTING RESULTS OF CANCER TREATMENT (1979). WHO Offset Publication, No. 48, WHO: Geneva.